# Analysis of length of hospital stay using electronic health records: A statistical and data mining approach

**DOI:** 10.1371/journal.pone.0195901

**Published:** 2018-04-13

**Authors:** Hyunyoung Baek, Minsu Cho, Seok Kim, Hee Hwang, Minseok Song, Sooyoung Yoo

**Affiliations:** 1 Office of eHealth Research and Businesses, Seoul National University Bundang Hospital, Seongnam, South Korea; 2 Department of Industrial and Management Engineering, Pohang University of Science and Technology, Pohang, South Korea; 3 School of Management Engineering, Ulsan National Institute of Science and Technology, Ulsan, South Korea; Yokohama City University, JAPAN

## Abstract

**Background:**

The length of stay (LOS) is an important indicator of the efficiency of hospital management. Reduction in the number of inpatient days results in decreased risk of infection and medication side effects, improvement in the quality of treatment, and increased hospital profit with more efficient bed management. The purpose of this study was to determine which factors are associated with length of hospital stay, based on electronic health records, in order to manage hospital stay more efficiently.

**Materials and methods:**

Research subjects were retrieved from a database of patients admitted to a tertiary general university hospital in South Korea between January and December 2013. Patients were analyzed according to the following three categories: descriptive and exploratory analysis, process pattern analysis using process mining techniques, and statistical analysis and prediction of LOS.

**Results:**

Overall, 55% (25,228) of inpatients were discharged within 4 days. The department of rehabilitation medicine (RH) had the highest average LOS at 15.9 days. Of all the conditions diagnosed over 250 times, diagnoses of I63.8 (cerebral infarction, middle cerebral artery), I63.9 (infarction of middle cerebral artery territory) and I21.9 (myocardial infarction) were associated with the longest average hospital stay and high standard deviation. Patients with these conditions were also more likely to be transferred to the RH department for rehabilitation. A range of variables, such as transfer, discharge delay time, operation frequency, frequency of diagnosis, severity, bed grade, and insurance type was significantly correlated with the LOS.

**Conclusions:**

Accurate understanding of the factors associating with the LOS and progressive improvements in processing and monitoring may allow more efficient management of the LOS of inpatients.

## Introduction

Length of hospital stay (LOS) is an important indicator of the use of medical services that is used to assess the efficiency of hospital management, patient quality of care, and functional evaluation. Decreased LOS has been associated with decreased risks of opportunistic infections and side effects of medication, and with improvements in treatment outcome and lower mortality rates. Furthermore, shorter hospital stays reduce the burden of medical fees and increase the bed turnover rate, which in turn increases the profit margin of hospitals, while lowering the overall social costs. [1, 2]

Previous studies have examined effective management of LOS. Majority of these involved subjects stratified by condition or admitting unit, for example, patients admitted to specialized departments, such as psychiatric wards [3] or the intensive care unit (ICU) [4, 5]; those with hip fractures[6] or undergoing coronary artery surgery[7]; or, those admitted with a specific diagnosis, such as heart failure [1, 8] or pulmonary disease [9].

LOS among patients with the same disease or undergoing the same type of surgical intervention may vary owing to complex factors related to the individual or due to different process flows within different organizations or divergences in medical practice. For these reason [10], in order to understand which factors are associated with LOS, all activities within the overall patient admission process flow should be analyzed from different perspectives.

In this study, electronic health records (EHR) data and process mining technology were used to analyze all event logs entered between admission and discharge of the patient. This allowed us to scrutinize issues regarding hospital processes that affect the actual LOS, as well as other related factors. The aim of this study was to determine a methodology that could be applied to help hospitals manage the duration of inpatient stay more efficiently.

## Materials and methods

### Data and preprocessing

Log data recorded between January and December 2013 were extracted from the EHR of a tertiary general hospital to analyze factors correlating with length of hospital stay. The subjects were patients admitted (and discharged) in 2013. The extracted event log is shown in Table 1.

**Table 1:** Types and attributes of event log data.

Event type	Attribute
Patient Information	Patient ID, Case ID, Diagnosis code (primary diagnosis), Diagnosis name (primary diagnosis), Department code (issued diagnosis), Severity, Insurance code, Hospital cost
Admission	Case ID, Indicated admission date, Scheduled admission date, Actual admission date, Department code, Physician ID, Bed grade
Surgery	Case ID, Surgery date, Surgery code, Surgery name, Surgeon ID, Surgeon’s department
Procedure	Case ID, Issued date, Performer ID, Performer’s department, Procedure code, Procedure name
Transfer	Case ID, Transferred date, Request date/Canceled date, Department code before transfer, Department code after transfer, Bed grade after transfer
Consultation	Case ID, Requesting physician ID, Requested physician ID, Department code, Consulting date, Answering date
Antibiotics	Case ID, Start date, End date, Antibiotics code, Antibiotics name
Discharge	Case ID, Indicated discharge date, Physician ID, Department code, Actual Discharge date

Case ID: a unique ID for identification of inpatients.

For the full year of 2013, we have collected 53,965 subjects except for 745 and 1,029 subjects who were in hospital at the first and last day of the year, respectively. Also, there was a lack of a discharge date for two subjects, and 8,295 patients were received the day surgery which does not have to be hospitalized. They were also removed from the set of target subjects being analyzed. In a nutshell, out of a total of 53,965 subjects, 8,419 subjects were excluded due to repeat admission for unexpected events (122), lack of a discharge date (2), and day surgery (8,295). Finally, data from 45,546 subjects were analyzed. For accurate data analysis, the following data were excluded: data presumed to have been wrongly entered, such as transfer note dates recorded before the admission date or after the discharge date; transfer completion dates recorded before the admission date; and procedures performed beyond the extracted date.

The present study was approved (IRB No. B-1409/268-107) by the Institutional Review Board of the Seoul National University Bundang Hospital, which waived patients’ informed consent. All EHR data provided to the researchers for this study were de-identified.

### Analysis methods

The methods of analysis used in the study are shown in Table 2 and categorized as: descriptive and exploratory analysis, process pattern analysis using process mining techniques, and statistical analysis and prediction for LOS. Descriptive and exploratory analysis seeks to understand from multiple angles, the current circumstances surrounding the hospital LOS. We included three detailed analysis items in this analysis category: performance analysis for LOS, LOS analysis according to diagnosis, and analysis for long-term hospitalization.

**Table 2:** Analysis methods.

Analysis Category	Analysis Items	Purpose & Method
Descriptive and Explorative Analysis	Performance analysis for LOS	■ Purpose: To comprehend the overall performance of LOS ■ Method: Analyzing basic data statistics using basic performance analysis [11] from process mining
	LOS analysis in accordance with diagnosis	■ Purpose: To determine the difference of LOS for each diagnosis ■ Method: Measuring the performance based on z-score [12] and conducting comparative analysis
	Analysis for long-term hospitalization patients	■ Purpose: To understand the features of long-term hospitalization patients and their differences from general in-patients ■ Method: Building clusters based on patients’ LOS and conducting comparative performance analysis
Process Pattern analysis using process mining techniques	LOS analysis in terms of transfer patterns	■ Purpose: To investigate the differences of LOS according to whether the transfer pattern was executed or not and each transfer pattern ■ Method: Discovering transfer patterns using pattern analysis [13] from process mining
Statistical analysis and prediction for LOS	Deriving correlated factors on LOS	■ Purpose: To understand the key factors that correlate with LOS ■ Method: Conducting statistical analysis using T-test and ANOVA [14]
	Building a predictive model of patients’ LOS	■ Purpose: To predict LOS of patients ■ Method: Building a prediction model using machine learning techniques such as regression analysis [15]

First, performance analysis for LOS uses the basis performance analysis technique[11] in process mining and measures basic statistics regarding the days spent in hospital. This included the analysis of the total distribution of hospitalization days, as well as the correlation between the number of patients in each department and the number of hospitalized days.

Secondly, LOS analysis according to diagnosis using Z-scores [12] to analyze differences in LOS by diagnosis. We postulated that analyzing the absolute LOS for all diagnoses was of limited value because there are considerable differences in the required LOS according to the specific diagnoses. In order to overcome this limitation, the relative LOS was measured and compared, by deriving the standard score of each diagnosis based on the mean and standard deviation of the LOS per department. This is demonstrated as follows(1) [12]:

$$Standard\;score\left(Z-Score\right)=\frac{observed\;LOS-expected\;mean\;of\;LoS}{standard\;deviation\;of\;LoS}$$ (1)

Analysis of long-term hospitalization aimed to determine the characteristics of long-term hospitalized patients. For this purpose, we used statistical analyses to observe patient clustering and applied performance analysis to each group, aiming to identify differences between the groups.

The second analysis category was process pattern analysis using process mining techniques to analyze the association between the transfer pattern and the number of hospitalization days. We used the pattern analysis technique [13] of process mining to extract the transfer pattern, and this technique offers performance information, such as the frequency of each pattern, the average time required, and the median time required.

The third analysis category is statistical analysis and LOS prediction identifying major factors correlating with LOS and predicting LOS based on the identified factors. For this purpose, statistical analysis techniques [14] included the Student’s *t*-test and Analysis of Variance (ANOVA), as well as machine running techniques [15], such as regression analysis.

Our approach has been analyzed with the ProM framework[16] and python open source scientific tools, Scikit-learn[17]. The ProM framework is the process mining open source software that provides plenty of analyzing functionalities. We have employed the basic performance analysis and the pattern analysis technique from the ProM framework. As far as the statistical analyses were concerned, we have utilized the Scikit-learn python module that provides a wide range of machine learning techniques.

### The LOS analysis framework

**Fig 1:**
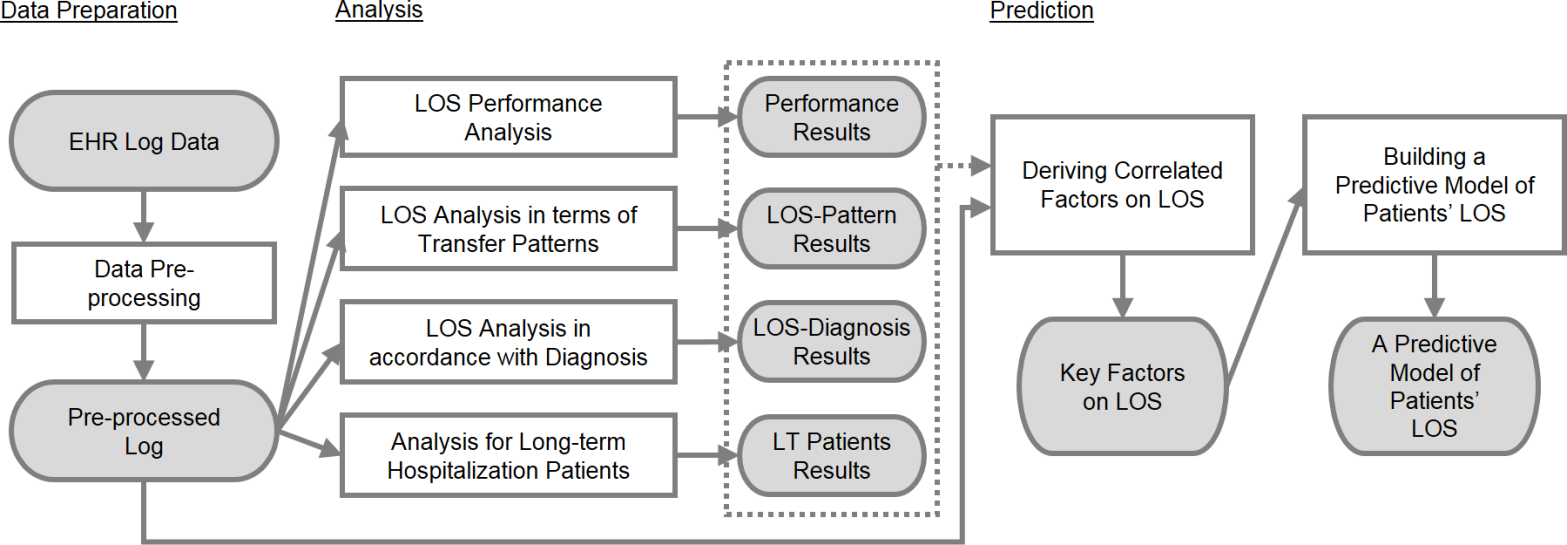
The LOS analysis framework.

In combining all the items above, using the EHR log data, we performed data pre-processing and analyzed data according to LOS. We also developed a LOS analysis framework which includes the overall flow of a model that can predict patient LOS. The LOS analysis framework is visually presented in Fig 1.

In the data preparation phase, we extracted the EHR log data. The data preprocessing process improved data quality in order to extract meaningful analysis results. In the data analysis phase, four types of analysis were performed: LOS performance analysis, LOS analysis of transfer patterns, LOS analysis according to diagnosis, and analysis of long-term hospitalization. The results of the analysis were meaningful as they revealed the association between LOS and other data items, and it also helped to understand the key factors correlating with duration of hospital stay at the prediction stage. At the prediction phase, the main factors correlating with the number of days of stay were identified through data analysis and log-based statistical analysis. A model was then developed to estimate the number of days of hospitalization based on the derived factors.

## Results

### Performance analysis for LOS

Examining the data from 2013, the hospitalized patients were averagely discharged around 7 days, and the range of the length of hospital stay was quite extensive (i.e., interquartile range: 2.0–8.0). The details for LOS-related values are provided in Table 3. Also, as far as the distribution of the LOS was concerned, approximately 55% (25,228) of hospitalized patients were discharged within 4 days, and out of these patients, approximately 20% (8,969) were left the hospital on the second day of hospitalization.

**Table 3:** Summary for length of stay of the hospitalized patients.

Metric	Value
Average	7.0
Median	4.0
Interquartile Range (i.e., IQR)	2.0–8.0
Minimum	0 (i.e., Patients were discharged on the same day)
Maximum	243.0

**Fig 2:**
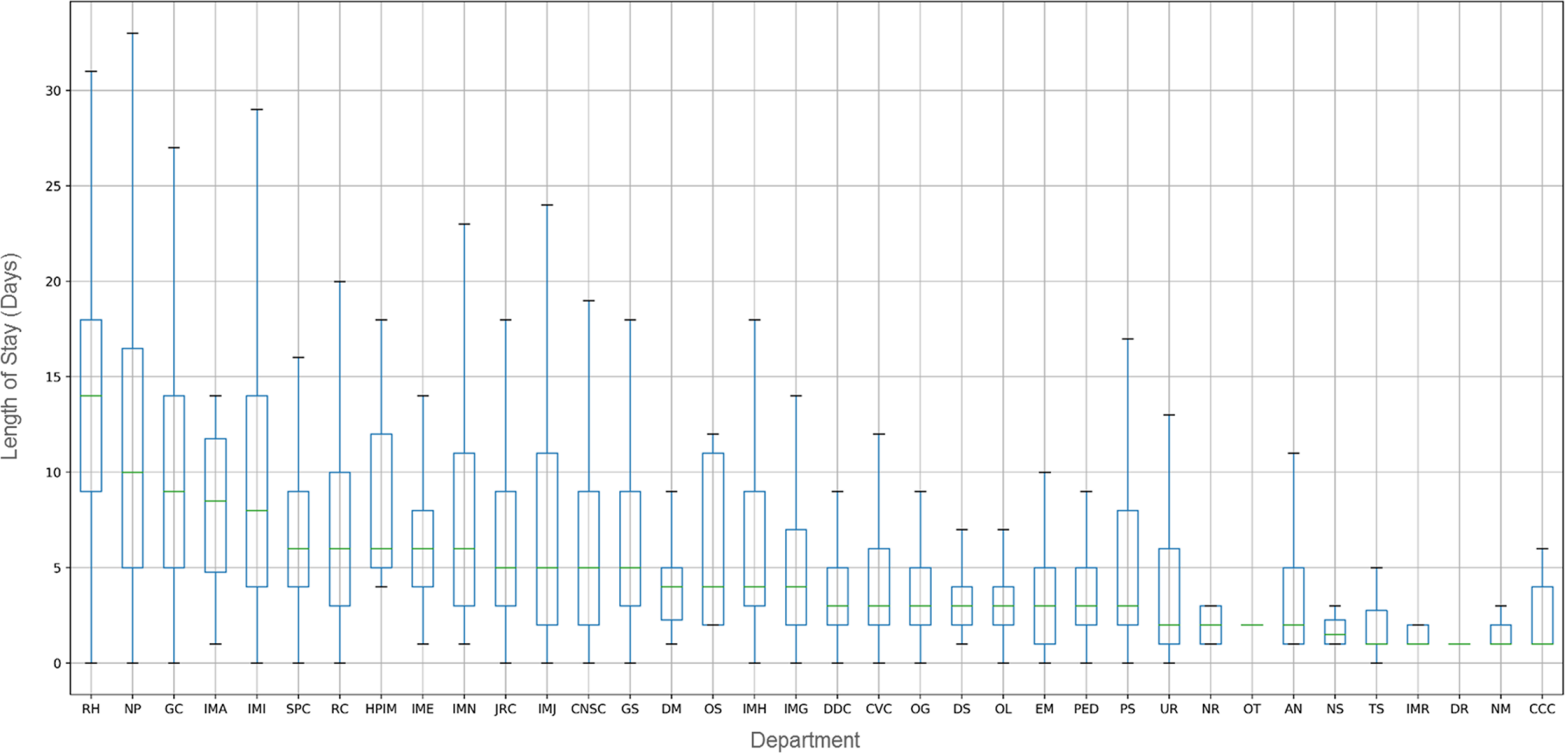
The distribution of length of stay. (a)Total length of stay. (b)Length of hospital stay by department.

Furthermore, a granular analysis on the length of stay was carried out by departments. Fig 2 presents the boxplot of the length of hospital stay for each department. Unexpected records, i.e., outliers, were removed in the graph for the effective comparative analysis.

The median length of hospital stay was 14 days in rehabilitation medicine; 10 days for neuropsychiatry; 9 days for geriatric center admissions; and 8 days for internal medicine, infectious diseases. Also, the IQR of hospital stay was 11.50 (i.e., 5.0–16.50) days for neuropsychiatry; and 10 (i.e., 4.0–14.0) days for internal medicine, infectious diseases.

**Fig 3:**
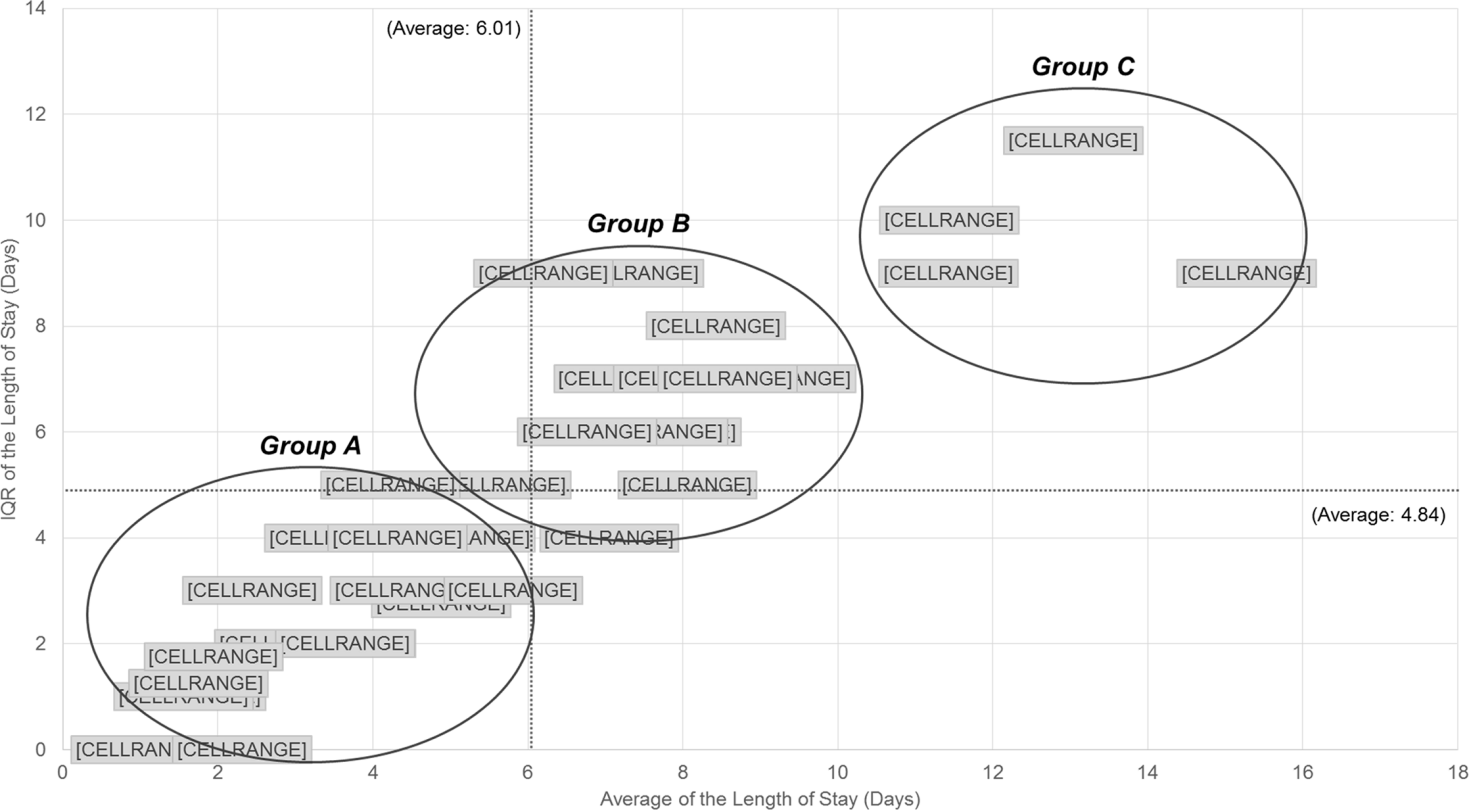
The results of the analysis according to the average and IQR of LOS per department.

Based on the analysis of average and interquartile range (i.e., IQR) of the LOS within each department, patients were divided into three groups. Note that IQR signifies the statistical dispersion of the distribution. Fig 3 shows the results of the analysis according to the average and IQR of LOS per department.

Based on the analysis for the average and IQR of LOS, we identified that there was a positive correlation between two measures. As it were, the average of LOS was higher, and the overall disperse of LOS was higher as well. Considering this trend, we classified the departments into three groups as follows.

- Group A–Average & IQR of the LOS: Low
- Group B–Average & IQR of the LOS: High
- Group C–Average & IQR of the LOS: Considerably High

First, *Group A* was the group with the relatively lower IQR and average LOS than other departments. The group included those being treated under radiology (DR), ophthalmology (OT), or obstetrics and gynecology (OG) among others. These departments within the group A were seen to be doing well in keeping their patients with the short and low dispersed stay. Therefore, it was judged to be a group with a considerably low need for improvement. *Group B* included relatively higher IQR and the average of LOS than other departments. Clinical neuroscience center (CNSC), internal medicine nephrology (IMN), and internal medicine allergy (IMA) exhibited this trait and were noted as the departments to be improved their inpatient management. It was identified that these departments had the average LOS close to the average of the whole departments, i.e., 6.01 days. However, some patients had remarkably higher LOS than others; thus, it resulted in the slightly high IQR value. Therefore, we concluded that there is a need to systematically manage the medical care process of the specific patients. Finally, *Group C* was characterized by significantly higher average and IQR of the length of hospital stay. Rehabilitation medicine (RH), neuropsychiatry (NP), internal medicine infectious disease (IMI), Geriatric Center (GC) were included in Group C, and detailed analysis of patient characteristics was required to identify issues that may cause prolonged LOS.

### LOS in accordance with diagnosis

**Fig 4:**
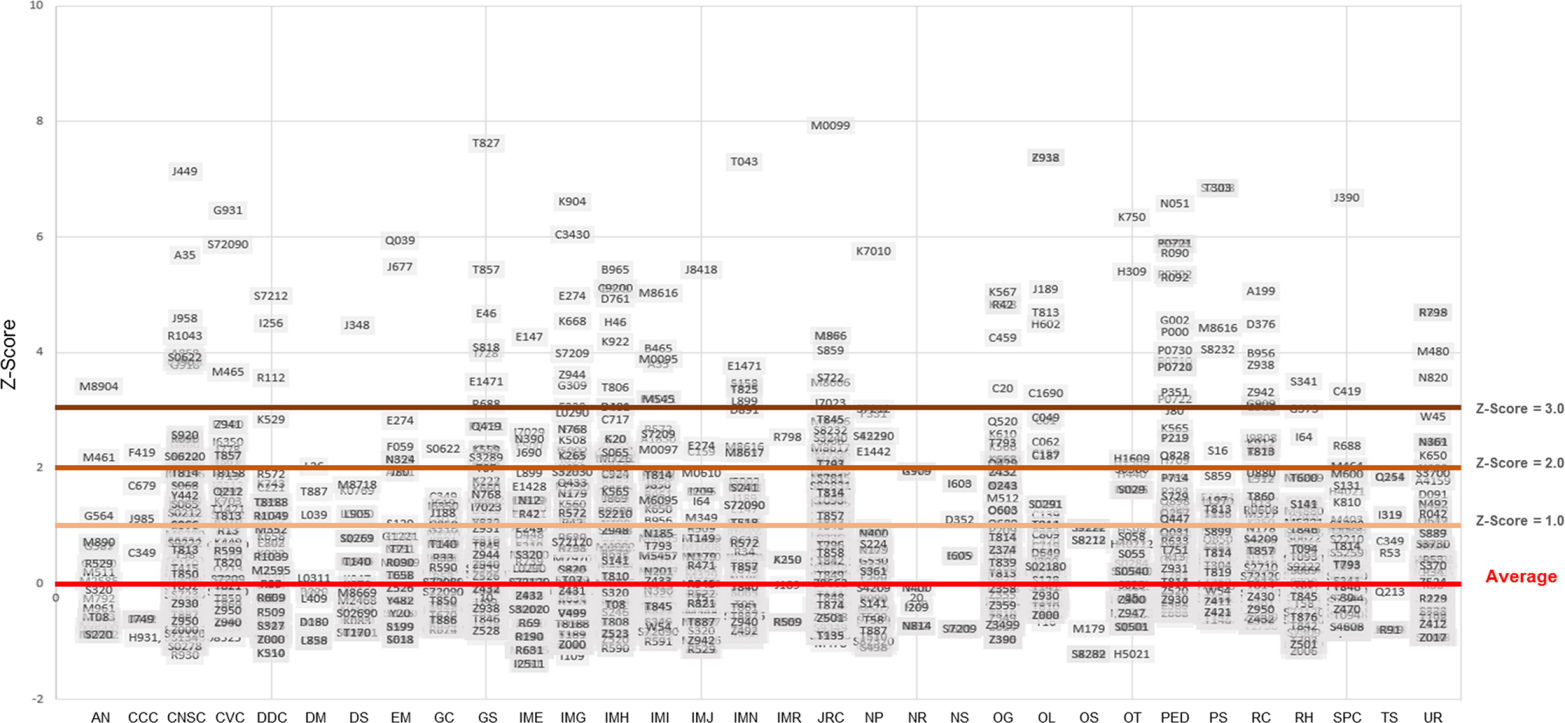
Diagnosis standard deviation distribution by department.

Diagnosis was a major factor correlating with the number of days of care [18]. LOS is determined by different variables and depends on specific diagnosis. The average LOS for each ICD-10 diagnosis issued by each department was converted to a Z-score, and then analyzed. An ICD-10 code consists of the first three characters for designating diagnosis category, the next three characters (characters three through six) for representing further details including the related etiology, anatomic site, or severity, and the seventh character for expansion. Fig 4 shows the distribution of diagnostic standard scores according to each department. Diagnoses such as J44.9(chronic obstructive pulmonary disease), T82.7(vascular graft infection), T04.3(crushing injuries involving multiple regions of lower limb), M00.99(septic arthritis site unspecified), Z93.8(jejunostomy state) often yield greater standard scores compared to other diagnoses, even within the same area of medicine.

Among high frequent diagnoses which were registered over 250 times, diagnoses of I63.8(cerebral infarction, middle cerebral artery) (Mean: 13.42 and IQR: 5–17) and I63.9(infarction of middle cerebral artery territory) (Mean: 13.96 and IQR: 5–19.5) were associated with a higher mean and interquartile range for LOS.

All two diagnoses above involved infarction and detailed analysis by medical care given showed that the patients were often transferred to RH for rehabilitation therapy, and there were also cases where multiple surgical interventions were performed on a specific patient. It is believed that such cases may have played a role in affecting the average LOS and also its interquartile range.

Table 4 shows the length of hospital stay for top 30 disease classification when all ICD-10 diagnosis codes were merged into the 3-digit level category. The diagnosis category of F30-F39(Mood [affective] disorders appeared to be the highest mean and interquartile range for LOS (Mean: 13.53 and IQR: 6–17.5) with moderate frequencies.

**Table 4:** Length of hospital stay by name of frequent disease classification (top 30).

	Classification of Diseases	# of Patients	Length of stay (days)				
			Mean	Med	IQR	Min	Max
C00-C97	Malignant neoplasms	6952	8.72	6	3–11	0	207
D00-D09	In situ neoplasms	5349	7.56	4	3–9	0	182
I60-I69	Cerebrovascular diseases	1934	12.50	7	4–15	0	207
K80-K87	Disorders of gallbladder, biliary tract and pancreas	1543	5.99	3	2–7	0	77
I70-I79	Diseases of arteries, arterioles and capillaries	1542	6.62	2.5	1–9	0	207
I20-I25	Ischaemic heart diseases	1177	4.08	2	2–4	0	213
N40-N51	Diseases of male genital organs	1073	3.36	1	1–4	0	72
D37-D48	Neoplasms of uncertain or unknown behaviour	1021	5.34	3	3–6	0	114
M60-M79	Soft tissue disorders	1007	8.03	6	4–9	0	116
Z40-Z54	Persons encountering health services for specific procedures and health care	965	3.69	3	2–4	0	28
M30-M36	Systemic connective tissue disorders	883	8.29	7	4–11	0	53
D10-D36	Benign neoplasms	869	3.73	2	1–4	0	69
O80-O84	Delivery	847	4.07	3	2–5	0	61
J20-J22	Other acute lower respiratory infections	677	9.10	6	4–10	0	114
K35-K38	Diseases of appendix	635	4.48	3	2–5	0	92
N80-N98	Noninflammatory disorders of female genital tract	564	3.59	3	3–3	0	65
I26-I28	Pulmonary heart disease and diseases of pulmonary circulation	530	5.56	2	1–4	0	142
J30-J39	Other diseases of upper respiratory tract	502	2.46	2	2–2	0	50
M80-M94	Osteopathies and chondropathies	484	9.66	5	5–9	0	243
J40-J47	Chronic lower respiratory diseases	474	2.91	2	2–2	0	88
R50-R69	General symptoms and signs	459	4.86	3	1–5	0	111
H65-H75	Diseases of middle ear and mastoid	407	3.92	3	3–4	1	35
E10-E14	Diabetes mellitus	396	4.40	4	3–4	1	45
G40-G47	Episodic and paroxysmal disorders	389	5.11	3	1–5	1	74
G50-G59	Nerve, nerve root and plexus disorders	386	5.11	3	2–5	0	87
S80-S89	Injuries to the knee and lower leg	383	6.11	4	3–6	1	128
N10-N16	Renal tubulo-interstitial diseases	380	7.04	5	3–9	0	92
F30-F39	Mood [affective] disorders	331	13.53	11	6–17.5	0	73
K55-K64	Other diseases of intestines	330	10.12	6	3–12	1	213
A15-A19	Tuberculosis	328	7.15	4	3–7	0	97

### Analysis for long-term hospitalization patients

Discharge of long-term inpatients is one of the main indicators actively managed by the hospital because shorter hospital stay is directly associated with an increase in hospital income, by increasing hospital turnover rate as well increasing the daily average cost of medical care. Usually, “long term inpatients” are defined as patients who have been hospitalized for over 30 days.

Patients were divided into three groups, according to LOS: A (under 7 days), B (7 days or more and under 30 days), and C (30 days or more). Approximately 3% (1,327people) of all patients were long-term inpatients in Group C. Table 5 shows that, compared to patients with shorter LOS, long-term inpatients included a significantly higher rate of surgical patients (54.26%), transferred patients (41.97%), and patients on antibiotic treatment (92.31%) as well as a greater number of surgical interventions (2.01 cases), antibiotics (116.55 cases), and procedures (385.99 cases) per patient, and a greater number of treatments per person per day (7.96 cases).

**Table 5:** Comparison between differing length of hospital stay.

Items	A (under 7 days)	B (7 ~ 30 days)	C (30 days or more)
Patient number (N) (percentage, %)	31,250 (69.00)	12,969 (28.00)	1,327 (3.00)
Average LOS (days)	3.03	12.23	48.51
Surgical patients (%)	38.72	50.75	54.26
Surgery per patient (cases)	1.01	1.13	2.10
Transferred patients (%)	0.75	12.35	41.97
Patient on antibiotics treatment (%)	54.98	77.86	92.31
Antibiotics per patient (cases)	7.57	24.78	116.55
Antibiotics per patient in a day (cases)	2.50	2.03	2.40
Procedures per patient (cases)	22.87	84.54	385.99
Procedures per person in a day (cases)	7.55	6.91	7.96

With increased LOS, patients are exposed to a higher risk of infections and the use of broad-spectrum antibiotics increases accordingly. The use of broad spectrum antibiotics may lead to the development of resistance to drugs and other serious side effects. For this reason, a number of antibiotics are managed as Restricted Antibiotics and subject to limited prescription. In this study, the ratio of restricted antibiotics administered to 1,000 randomly selected patients (12.79%) was higher than that in group A (0.7%) or group B (2.99%) (P-value <0.001).

### LOS analysis in terms of transfer patterns

Transfer was defined as a change of department required by the patient’s condition and one of the factors associating with the processing of hospitalized patients.

According to the analysis of hospital days based on transfer pattern, it was found that out of all patients, 5.25% (2,392) those who have been transferred on average spent 17 more days stay the hospital than those who were not transferred. There were highest number of incidents of patients being transferred to the departments of RH and IMH, and out of these, those being transferred from CNSC (Mean: 29.56 and IQR: 21.25–34.00) and SPC (Mean: 34.08 and IQR: 23.25–42.00) to RH had the highest interquartile range and also the average LOS. LOS by transfer pattern are shown in Table 6.

**Table 6:** Length of hospital stay by transfer pattern.

Items		# of patients	Length of stay (days)				
			Mean	Med	IQR	Min	Max
Transfer	Patients who were not transferred	43,154	6.08	4.0	2–7	0	243
	Patients who were transferred	2,392	23.12	17.0	10–29	1	213
Transfer patterns	Clinical Neuroscience Center (CNSC)->Rehabilitation Medicine (RH)	294	29.56	27	21.25–34	7	148
	Internal Medicine Gastroenterology (IMG)->General Surgery (GS)	251	16.73	12	8–20	2	110
	Respiratory Center (RC)->Internal Medicine Hematology (IMH)	169	11.33	9	7–13	3	67
	Joint Disease & Reconstruction Center (JRC)->Rehabilitation Medicine (RH)	71	25.08	22	18–28.5	4	87
	Spine Center (SPC)->Rehabilitation Medicine (RH)	62	34.08	28	23.25–42	11	88
	Internal Medicine Gastroenterology (IMG)->Internal Medicine Hematology (IMH)	55	11.55	9	8–12	3	44
	General Surgery (GS)->Internal Medicine Hematology (IMH)	46	15.33	11	8–19.75	3	59
	Clinical Neuroscience Center (CNSC) ->Internal Medicine Hematology (IMH)	41	14.93	13	9–18	3	56
	General Surgery (GS)->Plastic & Reconstructive Surgery (PS)	37	14.16	10	8–12	4	61
	Internal Medicine Nephrology (IMN)->Urology (UR)	22	13.59	10.5	7–20	6	31
	Internal Medicine Hematology (IMH)->General Surgery (GS)	22	18.27	16.5	10–24.75	5	43
	Cardiovascular Center (CVC)->Respiratory Center (RC)	21	19.48	17	10–24	7	63
	General Surgery (GS)->Internal Medicine Gastroenterology (IMG)	20	17.35	12	9–21.25	5	46

### Deriving correlated factors on LOS

Table 7 shows the results of the analysis between LOS and different hospitalization variables: time required for transfer, discharge delay, surgery frequency, diagnosis frequency, severity, bed grade, and insurance type.

**Table 7:** Variables associating with the length of hospital stay.

Item	Type	Number	%	Length of stay (days)						P-value
				Mean	Med	Std	IQR	Min	Max	
Transfer time ^1^^)^	less than 2 days	178	46.97	14.01	10	12.79	7–17	2	85	< 0.001
	2 or more days	201	53.03	23.99	18	23.28	11–27.75	4	205	
	Total	379	100.00	18.70	13	19.11	8–23	2	205	
Discharge delay time ^2^^)^	less than 1 days	38,166	83.80	6.96	4	9.94	2–8	0	243	0.031
	1–2 days	7,137	15.67	7.12	4	8.86	3–8	1	148	
	2 or more days	243	0.53	5.54	3	6.51	3–5	2	56	
	Total	45,546	100.00	6.98	4	9.76	2–8	0	243	
Surgery frequency	0	26,145	57.4	6.17	4	8.39	2–7	0	193	< 0.001
	1	18,286	40.20	6.97	4	8.41	3–8	0	197	
	2	933	2.00	21.25	15	20.02	10–24	1	205	
	3 or more	182	0.40	50.30	39.5	39.42	23–64	4	243	
	Total	45,546	100.00	6.98	4	9.76	2–8	0	243	
Diagnosis frequency	0	3	0.00	3.33	2	2.31	2–4	2	6	< 0.001
	1	41,696	91.55	6.07	4	7.63	2–7	0	243	
	2	3,477	7.63	14.53	9	16.50	4–20	0	197	
	3 or more	370	0.81	38.24	32	31.84	16–50	1	213	
	Total	45,546	100.00	6.98	4	9.76	2–8	0	243	
Severity	General	31,840	69.9	6.56	4	9.79	2–7	0	243	< 0.001
	Critical	13,706	30.1	7.94	5	9.62	3–9	0	197	
	Total	45,546	100.00	6.98	4	9.76	2–8	0	243	
Bed grade	Upper grade	24,870	38.17	2.90	2	3.98	1–3	0	99	< 0.001
	General	40,281	61.83	5.84	4	7.23	2–7	0	178	
	Total	65,151	100.00	4.72	3	6.35	1–5	0	178	
Insurance type	Health insurance	43,704	95.37	6.92	4	9.73	2–8	0	243	< 0.001
	Industrial accident	74	0.16	14.19	8	17.08	4.25–15	1	83	
	Medical assistance	1,192	2.60	8.99	6	10.56	3–11	0	120	
	Medical research	90	0.20	8.84	3	10.60	2–13	0	48	
	Self pay	418	0.91	5.52	3.5	6.48	2–6	0	47	
	Automobile	346	0.76	12.22	7	15.73	4–13	0	128	
	Total	45,824	100.00	7.01	4	9.82	2–8	0	243	

1) Department and ward transfer time taken: difference between the date of signing the transfer order and the date of transfer

2) Discharge delay time: difference between the date of discharge order and the date of discharge

Patients requiring 2 or more days to transfer had a greater number of hospital days (Mean: 23.99 and IQR: 11.00–27.75) than patient with a LOS of under 2 days (Mean: 14.01 and IQR: 7.00–17.00). Patient discharge time showed that patients requiring 1–2 days (Mean: 7.12 and IQR: 3.00–8.00) had a higher LOS than patients requiring 1 day or less (Mean: 6.96 and IQR: 2.00–8.00) or 2 days or more (Mean: 5.54 and IQR: 3.00–5.00). Hospital stay based on incidence of surgery showed that patients undergoing 3 times or more surgical interventions had the longest LOS (Mean: 50.30 and IQR: 23.00–64.00) compared to patients undergoing no surgery (Mean: 6.17 and IQR: 2.00–7.00), 1 intervention (Mean: 6.97 and IQR: 3.00–8.00) or 2 interventions (Mean: 21.25 and IQR: 10.00–24.00). In terms of diagnosis, patients with 3 or more diagnoses had the longest hospital stay (Mean: 38.24 and IQR: 16.00–50.00) compared to patients with no diagnosis (Mean: 3.33 and IQR: 2.00–4.00), 1 diagnosis (Mean: 6.07 and IQR: 2.00–7.00), 2 diagnoses (Mean: 14.53 and IQR: 4.00–20.00).

Patients receiving critical care (Mean: 7.94 and IQR: 3.00–9.00) were more likely to have longer LOS than those who were not (Mean: 6.56 and IQR: 2.00–7.00), and patients on general wards (Mean: 5.84 and IQR: 2.00–7.00) were more likely to remain in hospital longer than patients on upper grade wards (Mean: 2.90 and IQR: 1.00–3.00). Analysis of hospital stay according to insurance type indicated that admissions involving industrial accidents, medical assistance, medical research, and automobiles occurred less frequently than admissions on health insurance, although the LOS was relatively higher. All variables were statistically significant. (P<0.05)

### Building a predictive model of patient’s LOS

This section presents a model for the prediction of the number of days in hospital based on the significant variables analyzed above. Multiple regression analysis was performed to develop the model. The following five variables were used as independent variables: frequency of surgery, frequency of diagnosis, frequency of patient transfer, severity, and insurance type. LOS was used as a dependent variable. Also, we partitioned data into the training and test dataset to measure the accuracy of the model; 80% and 20% of data became the training and test data, respectively. Table 8 provides the result of the multiple regression analysis. All five variables were statistically significant and, therefore, correlated with the prediction of the length of hospital stay. In the regression model from the training dataset, *R*^*2*^ was 0.267, and duration of hospitalization was calculated as followed: LOS (days) = 2.72 + 2.70 * (frequency of surgery) + 2.46 * (frequency of diagnosis) + 11.65 * (number of transfer) + 1.02 * (severity)– 0.80 * (insurance type). As a result of measuring the accuracy with the test dataset, we identified that the mean absolute error of the model is 4.68.

**Table 8:** Length of hospital stay prediction model drawn.

Items	*β*	SE	t	P-value
Constant	2.72	0.29	9.50	< 0.001
Surgery frequency	2.70	0.07	39.70	< 0.001
Diagnosis frequency	2.46	0.16	15.22	< 0.001
Transfer frequency	11.65	0.20	57.20	< 0.001
Severity (Y = 1)	1.02	0.10	10.32	< 0.001
Insurance type (health insurance = 1)	-0.80	0.22	-3.59	< 0.001

Furthermore, we performed the further analysis for building a model to classify whether a specific patient is a long-term inpatient. As we said earlier, we defined long-term inpatients as who had the 30 or more length of hospital stays. As far as this analysis was concerned, random forest was employed to build a model, and data partitioning with 80% for the training and 20% for the test dataset was performed to measure the accuracy. As a result, it was found out that the accuracy of the classification model is 0.9732. That is, the model had the sufficient capacity to classify long-term hospitalized patients. Also, the relative importance of each feature, the transfer frequency was the was the highest at 41.40%, while surgery frequency, diagnosis frequency, severity, and insurance type were 28.44%, 24.13%, 4.77%, and 1.26%, respectively.

## Discussion

This study aimed to analyze the association between the EHR event data and LOS in inpatients using statistical analysis and process data mining analysis technology, in order to determine which factors are correlated with the LOS.

Among transferred patients, the mean length of hospital stay was increased by 17 days compared to those who were not transferred. The average number of hospital days (and standard deviation) was the highest among patients transferred from the CNSC and the SPC to RH. Furthermore, among ICD-10 diagnoses which have been registered more than 250 times, the most frequent three diagnoses were all related to infarction. A detailed analysis of medical care showed that majority of these patients were transferred to RH for rehabilitation. A similar distribution of patients by diagnosis was observed between patients transferred from the CNSC and from the SPC to RH.

Stroke, which accounts for the majority of cases of cerebral infarction, requires different rehabilitation treatment modalities, such as physical, speech, and occupational therapies, due to the wide variety of symptoms that occurs depending on the location of the damage in the brain. Although LOS was increased, increased rehabilitation time was associated with increased functional recovery. [19, 20]

When ICD-10 diagnosis codes are classified into the 3-digit level, one of the mental disorder, mood [affective] disorders showed the highest mean for the LOS. There have been studied the factors affecting the LOS in psychiatric patients.[21, 22] As psychiatric patients have different hospitalization factors according to personal characteristics, it seems to be needed to find effective ways of LOS management by analyzing various personal characteristics and treatment pathways among psychiatric patients.

Analysis of patient transfer and diagnostic data revealed that LOS was high among patients who were transferred to RH. It was plausible that secondary problems caused by the primary disease were more likely to correlate with the LOS. Clinical pathways (CP)[23], evidence-based and standardized practice guidelines are being developed and applied to improve the quality of medical care and to reduce the LOS, for both diseases and surgery. It may be necessary to have a standardized and optimized practice manual developed for secondary rehabilitation treatments (for function improvements) to be used in RH. It has been reported that the use of effective rehabilitation programs affects the cost reduction of hospitalization and also reduces the LOS. [24]

Long-term hospitalization exceeding 30 days was associated with a higher percentage of surgical operations and transfer rates, as well as restricted antibiotic use compared to other patients. It is very important to manage antibiotic use indicators to appropriately and effectively manage the quality of medical care.[25] While antibiotics are important in treatment and prevention of diseases, long-term use can lead to the development of drug resistance with serious effects on health. This can also increase the LOS and medical costs. [26] Therefore, antimicrobial usage should also be monitored and appropriately managed.

Furthermore, this study found that the following factors were correlated with LOS: transfer time, discharge delay time, frequency of surgery, diagnosis frequency, patient severity, bed grade, and insurance type. Most studies examining factors that are associated with the length of hospitalization involved subjects affected by specific diseases or types of surgery, limiting associated factors within the patients’ individual characteristics. For example, a study on pediatric asthma [27] found that variables correlating with patient LOS were age, sex, obesity, and chronic disease. In a previous study examining subjects who underwent radical cystectomy, sex, age, and complications were reported to be associated with hospital stay [28]. It is important to note that variables such as the patient characteristics cannot be improved by changes in hospital practice. This study considered all subjects admitted to hospital, rather than restricting participants according to medical condition or type of surgery. Therefore, the variables identified were those that could improve the overall processing and motoring system of the hospital.

Based on the significant findings of this study, strategies to improve the process of transfer and discharge in the entire hospital, were recommended. Moreover, it is anticipated that effective management of LOS is possible with a sustained effort to manage patients undergoing frequent surgery, with multiple diagnoses, and with severe conditions.

There were some limitations to this study which should be addressed. First, the analysis of patient process correlating with the LOS was based on data from a single hospital. As there are differences between hospitals in the admission process and treatment plans, generalizability was limited and it is important to collect and analyze data from multiple hospitals. Furthermore, data analysis was largely confined to the main hospitalization events of the EHR system; the general characteristics of the individual patients and the hospital’s environmental factors were not considered in the analysis. The LOS may also be related with month of the year or day of the week of admission/discharge date, for example, admissions on Friday not being discharged until Monday due to lack of senior staff on weekends.

Despite these limitations, this study analyzed the LOS based on objective EHR data that included all medical events for each inpatient rather than some specific patients. Importantly, this study is of value as it analyzed the factors correlating with LOS and identified solutions to reduce this time.

In future studies related to hospital stay, it may be necessary to collect multi-distribution data, as well as the general characteristics of individual subjects, their environmental factors and seasonal and date/time factors, which were not considered in this study.

## Conclusions

In this study, we analyzed different variables correlating with LOS by using EHR admission data. We considered how to improve the management of LOS among inpatients.

Research on the duration of hospital stay is important because it helps hospitals to more effectively manage its resources and patients. Specifically, identifying factors which are associated with the LOS in order to accurately predict and manage the number of inpatient days, could be helpful in terms of managing hospital resources and may enable the development of a Clinical Pathway useful for inpatient treatment.

Based on the variables identified in this study, it may be necessary to improve the financial structure of hospitals and develop institutional approaches to reduce patient medical fees, by promoting the effective use of hospital resources and reducing the length of hospital stay via a system subject to continuous monitoring. Eliminating unnecessary hospital stays is a strategy to reduce overall national medical expenses.
